# Arctic-like Rabies Virus, Bangladesh

**DOI:** 10.3201/eid1812.120061

**Published:** 2012-12

**Authors:** Khondoker Mahbuba Jamil, Kamruddin Ahmed, Moazzem Hossain, Takashi Matsumoto, Mohammad Azmat Ali, Sohrab Hossain, Shakhawat Hossain, Aminul Islam, Mohammad Nasiruddin, Akira Nishizono

**Affiliations:** Author affiliations: Institute of Epidemiology, Disease Control, and Research, Dhaka, Bangladesh (K.M. Jamil);; Oita University, Oita, Japan (K. Ahmed, T. Matsumoto, A. Nishizono);; Ministry of Health and Family Welfare, Dhaka (M. Hossain);; Dhaka City Corporation, Dhaka (M.A. Ali, S. Hossain, A. Islam, M. Nasiruddin);; Tongi Municipality, Tongi, Bangladesh (S. Hossain)

**Keywords:** rabies, Arctic-like rabies virus, rabies virus, viruses, zoonoses, molecular epidemiology, Bangladesh

## Abstract

Arctic/Arctic-like rabies virus group 2 spread into Bangladesh ≈32 years ago. Because rabies is endemic to and a major public health problem in this country, we characterized this virus group. Its glycoprotein has 3 potential *N*-glycosylation sites that affect viral pathogenesis. Diversity of rabies virus might have public health implications in Bangladesh.

Rabies virus causes severe encephalitis in a wide range of mammals, including humans. Conservative estimates suggest that 55,000 persons worldwide die of rabies each year (*1*). Although the case-fatality rate in humans is 100%, rabies is preventable by vaccination. Bangladesh has the world’s third highest death rate for human rabies, an estimated 2,100 deaths per year (*2*). Dogs are the main reservoir of the virus and are responsible for spillover infections in humans (*2*). Therefore, dogs should be the principal target for successful rabies elimination.

With political will and solid global epidemiologic information, rabies elimination is possible. Molecular typing of circulating rabies viruses is necessary to identify and develop effective control measures, and to understand the spread of certain rabies virus variants and their incursion into new regions (*3*). For rabies elimination, this knowledge is needed for establishing cooperative approaches between neighboring countries to which the disease is endemic.

Bangladesh is one of several countries in which no molecular study has been conducted to identify types of rabies virus circulating within its boundaries. A lack of knowledge of phylogenetic relationships of Bangladesh rabies virus with viruses in other countries continues to hinder coordinated rabies control efforts in the region. This study was conducted to characterize rabies virus circulating in Bangladesh and to determine its relationship with viruses in neighboring countries to clarify its epidemiologic relationships, origin, and transmission dynamics.

## The Study

Seven brain samples were collected from animals with suspected rabies in 3 districts of Bangladesh (Dhaka, Narayanganj, and Narshingdi) in 2010 (Table 1). A portion of brainstem was removed from each sample and preserved in TRizol (Invitrogen, Carlsbad, CA, USA) at –20°C. Total RNA was extracted from brain homogenate, cDNA was synthesized by using random hexamer primers, reverse transcription PCR was conducted to amplify gene fragments, and nucleotide sequencing of genes was performed (*4*).

**Table 1:** Characteristics of 7 animal samples tested for rabies virus, Bangladesh

Sample no.	Animal	Age, y	District	History	Signs and symptoms	GenBank accession no.*
BDR1	Dog	Unknown	Dhaka	Unknown	Angry, biting tendency, excessive salivation, gradually became drowsy	Not determined
BDR2	Cow	8	Narsingdi	Calf died of suspected rabies 1 wk earlier	Angry, salivation, drooping of tongue, inability to drink or eat	AB699208
BDR3	Cow	10	Dhaka	Unknown	Angry, salivation, frequent micturition, inability to drink or eat	AB699209
BDR4	Goat	3	Narayanganj	Dog bite 2.5 mo earlier	Angry, inability to eat and drink, biting tendency	AB699210
BDR5	Goat	2	Narayanganj	Dog bite to head 2 mo earlier	Angry, salivation, inability to eat and drink	AB699220 (whole genome)
BDR6	Cow	6	Dhaka	Unknown	Angry, salivation, trying to attack	AB699212
BDR7	Cow	5	Narayanganj	Dog bite 2 mo earlier	Angry, salivation, trying to attack	AB699213

*For glycoprotein gene.

Full-length nucleoprotein (N) and glycoprotein (G) gene sequences from samples were determined. Nucleotide identities of N and G genes were 98%–100%. Amino acid identities of N and G genes were 100% and 98%–100%, respectively. Complete genomic sequencing (11,928 nt) of strain BDR5 was also conducted.

Evolutionary analysis was performed by using full-length N gene. We created a maximum clade credibility phylogenetic tree using the Bayesian Markov chain Monte Carlo method available in BEAST version 1.6.1 (*5*). Analysis was conducted by using a relaxed (uncorrelated lognormal) molecular clock and a generalized time reversible + Γ + proportion invariant model (*6*). All chains were run for 90 million generations and sampled every 3,000 steps and an effective sample size >1,383 was obtained for all estimated parameters. Posterior densities were calculated with 10% burn-in and checked for convergence by using Tracer version 1.5 in BEAST.

The mean rate of nucleotide substitution estimated for the N gene was 2.3 × 10^4^ substitutions/site/year (95% highest posterior density [HPD] 1.4–3.1 × 10^4^ substitutions/site/year). This rate is consistent with that of a previous study (*7*). The phylogenetic tree showed that rabies viruses in Bangladesh belong to Arctic/Arctic-like group 2 (AAL2) (*3*) also known as Arctic-like-1 (*8*), in close association with the strain from Bhutan.

**Figure 1:**
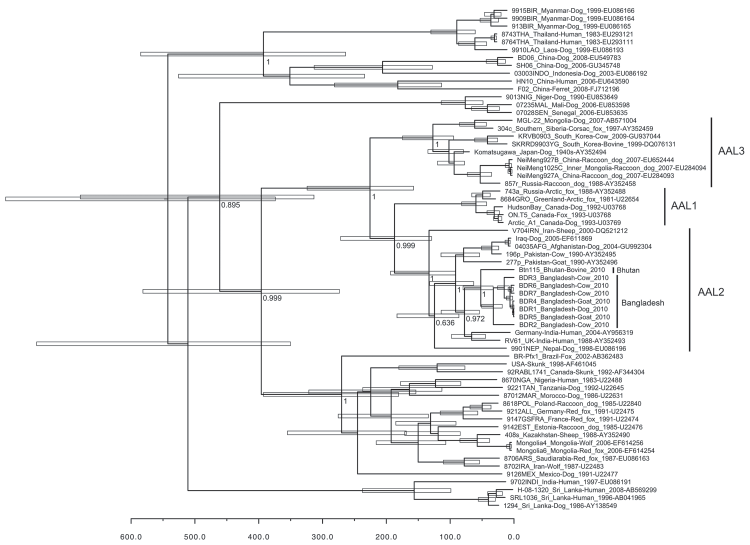
Bayesian maximum credibility tree showing genealogy of rabies virus obtained by analyzing nucleotide sequences of full nucleoprotein (N) gene sequences (1,350 nt), Bangladesh. Nodes indicate the mean age at which they are separated from the most recent common ancestor, and white horizontal bars at nodes indicate 95% highest posterior density values of the most recent common ancestor. Numbers at the main nodes indicate posterior values. Scale bar indicates time scale in years starting from 2010. Each strain name is followed by country of origin, host, year of detection, and GenBank accession number. Nucleotide sequence data of the N gene of rabies viruses from Bangladesh appear in the DDBJ/EMBL/GenBank nucleotide sequence databases: accession nos.: AB699214 (rabies virus strain BDR1), AB699215 (strain BDR2), AB699216 (strain BDR3), AB699217 (strain BDR4), AB699218 (strain BDR6), AB699219 (strain BDR7), and AB699220 (whole genome of strain BDR5). AAL, Arctic/Arctic-like.

Approximately 397.0 years ago (95% HPD 273.5–589.5 years), AAL and cosmopolitan rabies virus segregated from their most recent common ancestor (Figure 1). Approximately 225.6 years ago (95% HPD 157.4–324.2 years), AAL3 segregated. Approximately 187.4 years ago (95% HPD 129.0–271.9 years), AAL1 and AAL2 segregated. The AAL2 clade had a common progenitor that circulated ≈133.1 years ago (95% HPD 91.3–193.4 years), which has evolved into several different lineages. One lineage evolved 91.5 years ago (95% HPD 63.1–132.2 years) and currently circulates in Bangladesh, India, and Bhutan. Separate linages circulate in others countries in this region, including Iran, Nepal, Pakistan, and Afghanistan. AAL2 spread into central Bangladesh 32.3 years ago (95% HPD 18.4–50.6 years) in ≈1978 (95% HPD range 1958–1991).

Compared with the AAL2 strain from India (AY956319), BDR5 had several amino acid substitutions (Table 2). Sizes of their 2 genomes, leader RNA, trailer RNA, and intergenic regions were similar. The *N-*glycosylation site was predicted by using the NetNGlyc 1.0 server (www.cbs.dtu.dk/server/netnglyc). With the exception of BDR6, the G gene of all strains had potential glycosylation sites at position 37, 146, and 319.

**Table 2:** Substitutions in genome sequence of rabies virus BDR5 from Bangladesh compared with genome sequence of strain from India (AY956319)*

Protein, amino acid substitution	Site/domain/region of protein†
N	
Asp_378_ → Glu_378_	Antigenic site IV
Gln_422_ → Arg_422_	–
P	
Ser_64_ → Pro_64_	Variable domain I
Gln_71_ → Thr_71_	Variable domain I
Asn_90_ → Ser_90_	N protein binding site in variable domain II
Pro_159_ → Ser_159_	N protein binding site in variable domain II
His_162_ → Ser_162_	N protein binding site in variable domain II
Asn_166_ → Ser_166_	N protein binding site in variable domain II
Ala_174_ → Val_174_	N protein binding site in variable domain II
M	
Leu_21_ → Pro_21_	–
Ser_46_ → Gly_46_	–
Ile_168_ →Val_168_	–
G	
Ala_-(minus)15_ → Val_-15_	Signal peptide
Val_-(minus)6_ → Phe_-6_	Signal peptide
Val_7_ → Ile_7_	–
Asp_146_ → Asn_146_	Putative additional *N-*glycosylation: NKS
Val_427_ → Ile_427_	–
Arg_462_ → Gly_462_	Transmembrane domain
His_465_ → Arg_465_	Transmembrane domain
Gly_473_ → Ser_473_	Transmembrane domain
L	
Asp_18_ → Glu_18_	–
Ala_19_ → Thr_19_	–
Arg_315_ → Lys_315_	Conserved domain I
Val_361_ → Leu_361_	Conserved domain I
His_640_ → Gln_640_	Conserved domain III
Lys_657_ → Arg_657_	Conserved domain III
Ala_966_ → Thr_966_	Conserved domain IV
Pro_1133_ → Ser_1133_	Conserved domain V
Arg_1307_ Lys_1307_	Conserved domain IV
Asp_1373_ → Gly_1373_	–
Leu_1626_ → Val_1626_	–
Leu_1654_ → Ser_1654_	–
Val_1755_ → Ile_1755_	–
Cys_1825_ → Tyr_1825_	–
Asn_1841_ → Lys_1841_	–
Gln_1845_ → His_1845_	–
Cys_1872_ → Phe_1872_	–
Asn_2091_ → Ser_2091_	–

*N, nucleoprotein; P, phosphoprotein; M, matrix protein; G, glycoprotein; L, polymerase. †– indicates that the amino acid substitution was in a location that has no site/domain/region name. NKS, asparagine-lysine-serine.

## Conclusions

Genetic analysis and phylogenetic studies can contribute to understanding the epidemiology of rabies virus in disease-endemic countries. Molecular analysis of animal rabies viruses showed that AAL2 appeared in central Bangladesh only 32 years ago. A close association between N genes sequences from rabies viruses in Bangladesh and Bhutan indicates that they originated from a common ancestor. If one considers the ease of human movement between countries, AAL2 most likely entered Bangladesh from India rather than from Bhutan.

**Figure 2:**
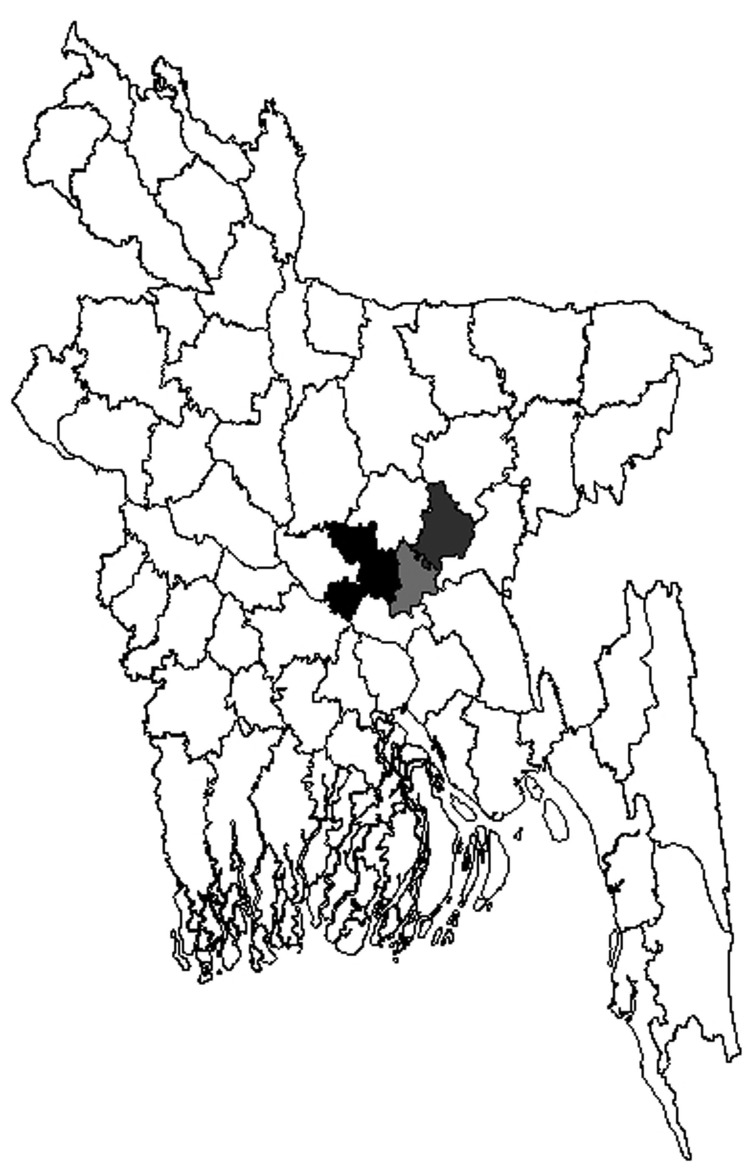
Three districts of Bangladesh from which samples were tested for Arctic/Arctic-like rabies virus and strains were found. Black, Dhaka District, strains BDR1, BDR3, and BDR6; light gray, Narayanganj District, strains BDR4, BDR5, and BDR7; dark gray, Narshingdi District, strain BDR2.

Circumstantial evidence suggests that rabies virus spread from India to Bhutan (*9*). AAL2 circulates in many states of India. It has spread into southern India and has replaced older strains (*10*,*11*). It is likely that AAL2 is also circulating in states of India that are between Bhutan and Bangladesh. Estimated time of AAL2 spread is based on 7 samples that are representative of central Bangladesh (Figure 2). Therefore, further surveillance might identify the extent to which AAL2 has spread and the diversity of rabies viruses in other parts of Bangladesh that might alter the estimated date of spread. It has been reported that arctic rabies virus and other variants can co-circulate in the same region (*12*).

The G protein is the major factor responsible for the pathogenesis of rabies virus and contains 2 glycosylation sites (*13*). The G protein of strains from Bangladesh uniquely evolved to contain 3 potential glycosylation sites, which has been reported in only fixed (laboratory adapted) strains and proposed to be responsible for their reduced pathogenicity (*13*). However, the site for additional glycosylation differs between Bangladeshi and fixed strains. Detection of an additional glycosylation site and amino acid substitutions deserve further investigations.

AAL viruses could have moved southward from Siberia or other northern regions of the former Soviet Union into Nepal, India, and other countries in Asia by a species jump from fox to dog at some point (*3*). Another possibility is that AAL viruses first emerged in dogs in southern Asia and subsequently spread to northern climes, where they are now maintained in fox populations (*3*,*8*). Extensive surveillance of viruses from Iran, Iraq, Afghanistan, and countries north of them is necessary to determine the origin and spread pattern of AAL rabies virus.

The timeline of divergence of different lineages determined in this study was similar to that previously reported (*8*). That study and our study used the full-length N gene to determine the time of divergence. Another study reported the timeline of divergence as a more recent event (*14*). This study used partial sequences of N genes, which might be responsible for different results. Rabies virus from Nepal also belongs to AAL2, and as reported in a previous study (*15*), seemed to be forming a different lineage. However, the speculation was not supported by a significant a posterior density value (0.6355). Thus, a network of countries is urgently needed to exchange information on molecular typing of circulating strains of rabies virus that might be useful in controlling rabies in this region.
